# Accuracy of menu calorie labelling in the England out-of-home food sector during 2024: assessment of a national food policy

**DOI:** 10.1017/S0007114525105217

**Published:** 2025-10-28

**Authors:** Amy Finlay, Andrew Jones, Paula Thorp, I Gusti Ngurah Edi Putra, Megan Polden, Jean Adams, Jane Brealey, Eric Robinson

**Affiliations:** 1 Department of Psychology, University of Liverpool, Liverpool, UK; 2 School of Psychology, Liverpool John Moores University, Liverpool, UK; 3 Department of Public Health, Policy and Systems, University of Liverpool, Liverpool, UK; 4 Department of Primary Care and Mental Health, University of Liverpool, Liverpool, UK; 5 NIHR Applied Research Collaboration Northwest Coast, Liverpool, UK; 6 MRC Epidemiology Unit, University of Cambridge, School of Clinical Medicine, Cambridge, UK

**Keywords:** Calorie labelling, Food policy, Accuracy, Out-of-home

## Abstract

Mandatory calorie labelling was introduced in out-of-home (OOH) food sector outlets during 2022 in England. Previous research in North America has found that labelled energy content can be underestimated for packaged and quick-serve foods, but no study has evaluated the accuracy of OOH food sector menu calorie labelling in response to the mandatory policy introduced in England. *N* 295 menu items from a range of outlet types (e.g. cafes, pubs, restaurants) and menu categories (e.g. starters and sides, main, dessert) were sampled. Bomb calorimetry was used to quantify energy content, and the reported energy content on menus was recorded. Consistency of measured energy was assessed by sampling the same items across outlets of the same business (*n* 50 menu items). Differences between reported and measured energy content were tested through Wilcoxon signed rank tests, and a linear model examined correlates of the difference. Mean measured kilocalories (kcal) were significantly lower than reported kcal (–16·70 kcal (±149·19), *V* = 16 920, *P* < 0·01 and *r* = 0·182). However, both over- (23 % of menu items) and under-estimation (11 %) by > 20 % of measured energy content were common, and the averaged absolute percentage difference between reported and measured values was 21 % (±29 %). Discrepancy between measured and reported energy content was more common in some outlet types (pubs), and reported energy content was substantially different (> 20 %) to measured energy content for 35 % of sampled menu items. There may be significant inaccuracies in reported energy content of calorie labelled menu items in English food outlets subject to mandatory calorie labelling.

Consumption of food prepared out-of-home (OOH), for example from cafés, restaurants and fast-food outlets, is associated with increased energy consumption^(1)^ and poorer dietary quality^(2)^. It has been estimated that in the UK, 60 % of the population purchase OOH food weekly, with the OOH food sector contributing on average 11 % of weekly energy intake^(3)^. In April 2022, calorie labelling on food menus was made mandatory in England for all businesses with over 250 employees. This policy was specific to the OOH food sector and applied to all non-prepacked foods and drinks made for immediate consumption^(4)^. The aim of the mandatory calorie labelling policy was to promote informed food choices and healthier eating behaviour in the OOH food sector^(5)^. Calorie labelling policies have been implemented outside of the UK, specifically in parts of Australia^(6)^, Canada^(7)^ and in the USA nationally^(8)^. Evidence is mixed on the impact that calorie labelling has on consumer behaviour^(9,10)^, but studies tend to suggest that calorie labelling results in reductions in energy content of menu items^(11,12)^.

Implementation guidance for the 2022 mandatory calorie labelling policy in England^(5)^ states that businesses can calculate the energy content of menu items by various means. Although laboratory analysis is considered the scientific gold standard, this is expensive and may not be feasible for many businesses. Energy content can be estimated using established and accepted food databases (for example, the McCance and Widdowson’s Composition of Foods Dataset), where nutritional composition is based on standard recipes^(13)^. A limitation of this method is that values may not be accurate for recipes that deviate from this standard. Energy values can be averaged based on manufacturer’s analysis or calculated using known or average values of ingredients used and imputing them into nutritional calculators such as MenuCal^(5)^. However, often these approaches will not account for cooking method used, which has a meaningful impact on the final served energy content.

A 20 % discretion between the reported and calculated energy content is permitted and necessary to provide businesses flexibility to account for differences in ingredient composition, portion size or calculation methods. Guidance acknowledges that accurate testing of energy content may not be viable for local enforcement officers^(5)^. Regarding enforcement, this guidance states that ‘Local authorities have discretion in how they enforce the Regulations’^(5)^. Specific roles of the enforcement officer include checking that calorie labels are present and that the methods used to estimate energy content are appropriate^(5)^. However, there is little assistance for enforcement officers in assessing the accuracy of calorie labelling, and no explicit instruction or suggestion that accuracy of labelling of menu items should be tested.

In the USA, businesses appeared initially to have largely complied with the mandatory provision of calorie labelling, with 79 % of businesses fully or partially implementing labels in advance of policy enforcement,^(14)^ and similarly in the UK, 80 % of businesses provided calorie labelling at any point of choice 6 months post-implementation, compared with only 21 % who provided this information prior to implementation^(15)^. The food industry has a history of inadequate compliance and exploitation of loopholes in government policy^(16–18)^. This is likely due to the lack of accountability by the food industry surrounding implemented policy and the lack of resources for compliance monitoring^(19)^.

There is evidence that nutritional labels can be prone to some degree of inaccuracy for packaged food products. One study found that the energy content of popular USA snacks was on average higher than reported on labels^(20)^. However, for most items (96 %), reported energy was within 20 % of measured energy content. In Canada, over 1000 items from supermarkets, bakeries and restaurants were tested for their nutritional content^(21)^. For the tested foods, sodium and energy were consistently under-reported. For 14 % of foods, the measured energy content exceeded label values by more than 20 %. A study conducted in the USA assessed the accuracy of nutrition labels for low-energy (< 500 kcal) restaurant and frozen meals^(22)^. For frozen meals, measured energy content was on average 8 % higher than reported, and for restaurant meals, energy content was on average 18 % higher than reported. Several restaurant foods contained up to twice the reported energy content. Similarly, a USA study of restaurant menu items in 2011 found that although average measured *v*. reported energy content across menu items was similar, 19 % of individual menu items had a measured energy content of ≥ 100 kcal per portion more than reported^(23)^.

No research has to date assessed the accuracy of calorie labels in OOH outlets following the implementation of the mandatory calorie labelling policy in England. As customers are encouraged to pay attention to the nutritional labelling of foods^(4)^, it is expected that the provided information will impact behaviour, and inaccurate nutritional labelling may hinder individual efforts to eat healthier. For example, if consumers use calorie labels to factor foods into their daily energy allowance, then underestimation of calories on food menus would lead to consumers unknowingly consuming excess energy. Additionally, if consumers identify potential inaccuracies in labelling themselves, then they may lose trust and stop using calorie information in the OOH food sector. Furthermore, some research suggests that there is little or no enforcement of the policy requirement of providing accurate calorie information^(24)^. Therefore, in the present study, we examined the accuracy of menu calorie labelling in OOH sector outlets subject to the 2022 mandatory calorie labelling law in England. As ingredient and cooking method information from businesses is not publicly available, laboratory analysis of menu items’ energy content was used to assess accuracy of menu calorie labelling in the present research.

## Materials and methods

This observational study was pre-registered on the Open Science Framework https://osf.io/8tfu4/.

### Outlet selection

Outlets in two local authorities (LA) in England were sampled to ensure findings were not area specific. Liverpool (North of England) and Milton Keynes (South of England) were selected to ensure mixed geographical coverage and representation of different quintiles of deprivation (index of multiple deprivation (IMD) used at the LA level). LA IMD quintiles (1–5) were used as a local measure of deprivation with IMD1 reflecting the most deprived areas and IMD5 reflecting the least deprived. LA selected represent quintile 1 (most deprived – Liverpool) and quintile 3 (medium deprivation – Milton Keynes). However, outlets in both LA were located across quintiles 1–5 as measured at the Lower Super Output Area.

Previous research that evaluated the impact of the calorie labelling legislation on consumer and business behaviour^(25)^ used the Inter-Department Business Register to identify businesses likely to be subject to the mandatory calorie labelling policy within LA of interest (data sampled June 2021, list produced Autumn 2020). The Inter-Department Business Register is a list of all UK businesses, their core characteristics, number of employees and principal activities defined using the Standard Industrial Classification. Standard Industrial Classification codes likely to include businesses serving food were identified (see the full list of Standard Industrial Classification codes in online Supplementary Material 1). Large businesses (defined as 250+ employees) within the relevant classification codes were then located, and their individual outlets were identified using Ordnance Survey Points of Interest data from September 2020. We used the final list of eligible businesses to guide outlet selection in the two LA.

**Figure 1.**
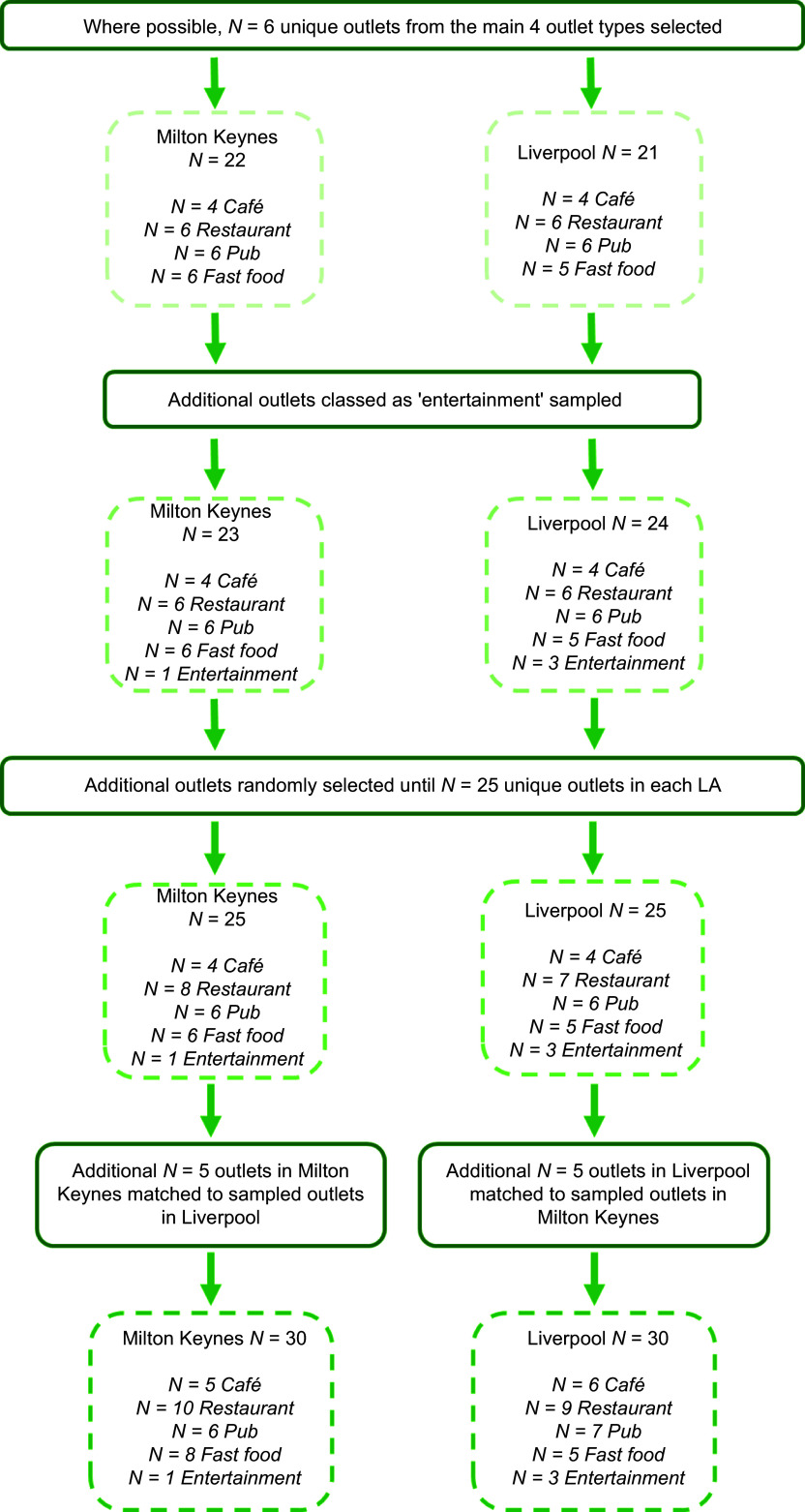
Outlet selection process.

Through power calculations, we deemed that a minimum of *n* 239 samples (menu items) (*n* 240 rounded to the nearest multiple of 2) would be sufficient to detect small effect sizes through paired samples tests in our primary analysis (see online Supplementary Material 2 for further detail). To obtain 240 samples with four samples per outlet (not including items deemed potentially inaccurate, which were analysed separately) required sixty outlets in total or thirty outlets in each LA. For a sub-sample of outlets (*n* 5 in each LA), we planned to match outlets across LA and conduct paired sample comparisons to assess consistency within chains. Figure 1 depicts the outlet selection process. To obtain a sample with variation in outlet type and item type, we randomly selected (using the RAND function in excel) *N* 6 unique outlets from each of four main outlet categories: café, restaurant, fast food and pubs. These outlet categories were taken from previous research evaluating the national calorie labelling policy^(26)^. If for any outlet category there were not six unique outlets (i.e. there were only multiple outlets from the same chain), additional outlets classed as ‘sport & entertainment’, for example, cinemas, were sampled. We randomly selected additional outlets from the full database (irrespective of outlet type) until *n* 25 unique outlets were selected for each LA. Duplicates of businesses across the two LA were permitted.

To assess consistency in calorie labelling between the same businesses across the two local authorities, *n* 5 (20 %) selected outlets in each LA were matched to an additional corresponding outlet from the same business in the other LA where the same items would be sampled. This resulted in an additional *n* 10 outlets sampled and a total of *n* 50 matched pairs for our planned secondary analyses. Additional outlets could only be sampled if they were not already selected. For example, if the randomly selected outlet in LA1 was a Starbucks, an additional matched Starbucks would be identified in LA2 for sampling. In total, we sampled *n* 300 menu items from sixty outlets (thirty in each LA). For full sample size and power analysis information, see online Supplementary Material 2.

### Menu item selection

Prior to data collection, online menus for each outlet were used to identify the items on offer in outlets. For each outlet, one menu item was selected for sampling in each of the following categories:

- Starter/side or prepared drink (randomly selected)
- Main meal (randomly selected)
- Dessert (randomly selected)
- Menu item that that was potentially inaccurate (determined by a research staff member and trained nutritionist assessing the full menu)
- Most popular menu item (determined by asking serving staff on the day of data collection)

In instances where menu categories were not distinct (e.g. starter – main – dessert – side dish), two researchers individually considered the categories provided by outlets and re-categorised items into the above groups. Any differences were resolved through discussion. For some outlets, specifically cafés, the categories outlined above did not encompass the majority of items available, and so prepared drinks were sampled in place of starters/sides. This was the case for *n* 7 outlets from *n* 3 unique businesses.

Menu items categorised as starter/side or prepared drink, main meal and dessert were numbered, and a random number generator was used to select items for sampling prior to data collection. To determine items sampled under the category of potentially inaccurate, two researchers considered the available description of menu items and whether the reported energy content looked inaccurate using menu information alone (either too high or too low). We adopted this approach to reflect how enforcers of the policy may be expected to assess the accuracy of calorie labels on food menus (i.e. relatively subjectively). If there were no items that appeared to be inaccurate at face value (e.g. very high or low energy content based on type of menu item), researchers considered instances where potential variability in serving size (e.g. inconsistency in the serving size of a dish of pasta) could result in differences in energy content from reported. Once each researcher had determined a potentially inaccurate item for each outlet, a final item was decided upon through discussion. Due to the absence of sales data to assess popularity, in each outlet, the researcher asked the serving staff what the most popular item on the menu was, and this item was sampled. If the most popular menu item or potentially inaccurate item from an outlet was a drink, this was eligible for sampling.

For instances where menu items required customisation (e.g. choosing a side to go with a dish), researchers randomly selected from all available customisation options.

### Energy content of sampled items and procedure

The researcher responsible for data collection recorded the energy content reported on menus for each sampled menu item on the day of sampling. Samples were collected (Monday–Thursday) between 11.00 and 19.00 during April-May 2024. For each outlet, the researcher responsible for data collection ordered food to dine in. All sampled items (*n* 300 from *n* 60 outlets) were individually weighed and packaged in the restaurant. Calibration weights were used to ensure scales were accurate. All items were sent via a courier to an external laboratory accredited by the UK Accreditation Service (SGS Cambridge) for nutritional analysis through bomb calorimetry, and energy (kcal) data were analysed.

### Analysis

#### Primary analyses

For all analyses, the potentially inaccurate items were considered separately from all other items tested as we anticipated their inclusion may overestimate average difference between measured and reported energy content. For the main analyses, one item was missing due to lab error (spoiled food due to storage error), and three items were excluded from analysis due to implausibility of energy values. This resulted in *n* 236 samples. Similarly, for potentially inaccurate items, one menu item was missing due to lab error, resulting in *n* 59 samples.

Throughout the results, relative differences are calculated as *measured kcal – reported kcal* for both mean and percentage difference. Absolute differences are calculated as the mean or percentage difference from measured kcal regardless of the direction of the difference, calculated using the ‘abs()’ function in R Studio.

Absolute percentage differences were used to explore whether differences between measured and reported energy content differed from 0 % (perfect accuracy) and 20 % (permitted inaccuracy). As data were not normally distributed, one-sample Wilcoxon signed rank tests were conducted against 0 and 20 % to examine if the observed absolute difference values were significantly different from 0 % and 20 %.

We also conducted Wilcoxon signed rank tests to assess whether there was a significant difference between reported energy and lab-measured energy content (relative difference).

#### Secondary analyses

For the subsample of restaurants where a corresponding outlet from each LA was tested, resulting in *n* 50 matched pairs, we examined whether the measured energy content differed between the two locations using a paired Wilcoxon signed rank test. We created a new variable (measured kcal – reported kcal) and fitted a linear regression model to examine potential predictors (e.g. outlet characteristics) of the relative difference between measured and reported energy. We explored outlet type, menu category, item energy content, IMD score for outlet and LA. If any predictor variables were significant, we conducted subgroup analyses to explore differences. We also used this method to explore predictors of absolute mean percentage difference.

Results for primary analyses were considered significant at *P* < 0·05. To account for multiple comparisons, results for secondary and any exploratory analyses were considered significant at *P* < 0·01. For explanation of deviation to the pre-registered analysis plan, see online Supplementary Material 3.

## Results

**Figure 2.**
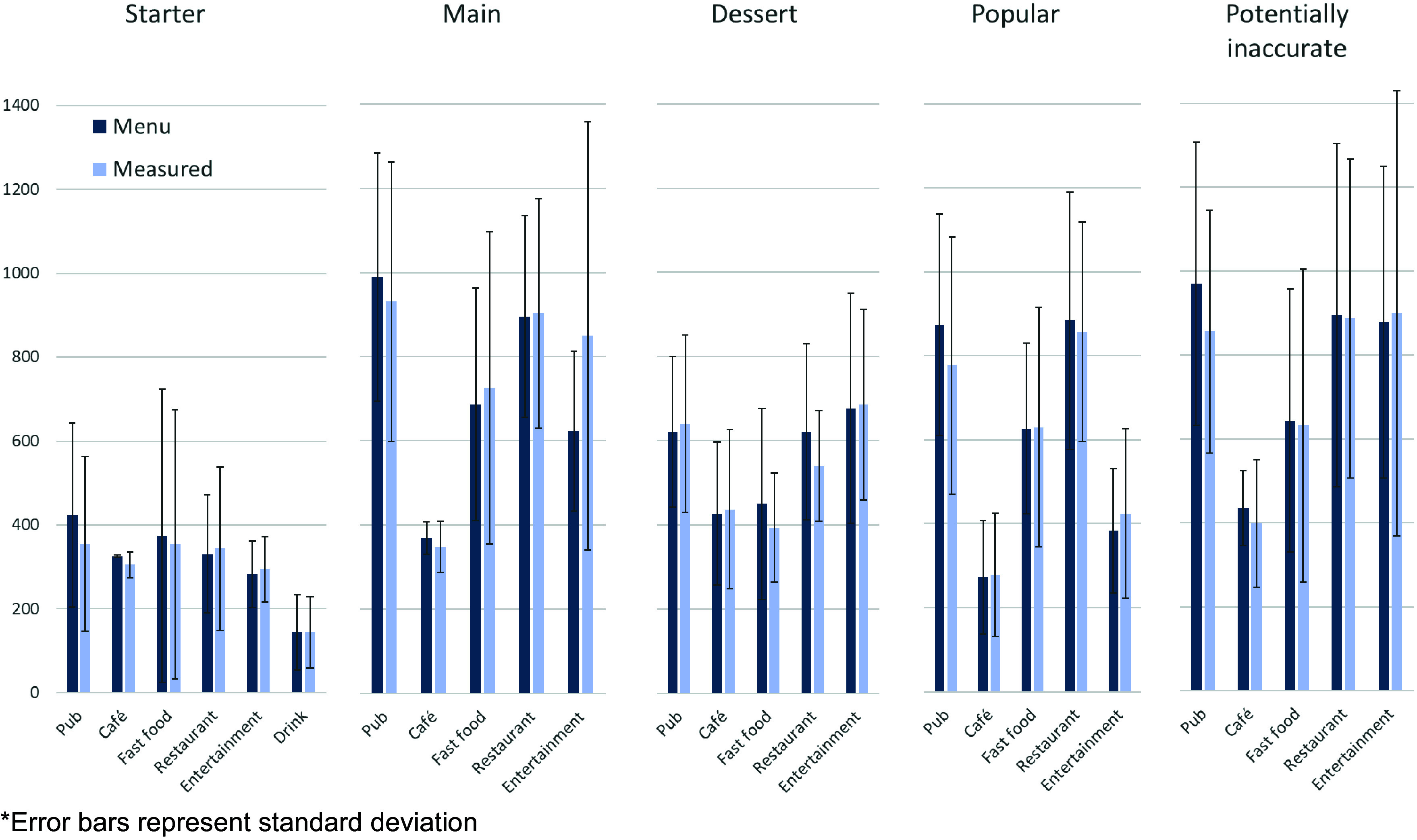
Reported *v*. Measured energy (kcal) split by outlet type and food category. *Error bars represent standard deviation.

A total of *n* 295 menu items were sampled from *n* 60 outlets across two LA in England. Figure 2 displays the mean reported and measured energy content for each category of item.

Menu items were categorised by outlet type (pubs, cafes, fast food, restaurants and entertainment) and menu category (starter/side, main, dessert, popular item, potentially inaccurate item and drinks). Mean energy and percentage differences between reported and measured energy for each category are reported in Table 1. For the most popular menu items and items that were potentially inaccurate, approximately 2/3 of sampled items were main meal items. Overall, the mean relative difference between reported and measured energy (measured-reported) was –16·70 kcal (±149·19) and −8·75 % (±35·27 %) expressed as a relative percentage of the menu item energy content. This was 98·76 kcal ±112·88 and 21·30 % (±29·45 %) when the absolute energy and percentage difference were examined (i.e. mean difference regardless of direction of difference).

**Table 1.** Difference between reported and measured energy (kcal) for the different categories

	Mean kcal content	Relative mean difference (measured-reported) (kcal)^*^	Relative percentage difference^†^	Absolute mean difference (kcal)^‡^	Absolute percentage difference^‡^	Percent of items outside 20 % leeway
Overall (*n* 236)	560·40 (329·11)^§^	–16·70 (149·19)	–8·75 % (35·27 %)	98·76 (112·88)	21·30 % (29·45 %)	34·75 %
Outlet category						
Cafes (*n* 44)	313·03 (158·74)	–2·60 (48·96)	–3·44 % (20·52 %)	36·29 (32·51)	15·02 % (14·30 %)	29·55 %
Pubs (*n* 51)	674·00 (338·37)	–51·00 (154·94)	–20·08 % (50·54 %)	126·26 (101·97)	29·78 % (45·46 %)	45·10 %
Fast food (*n* 51)	523·75 (323·79)	–8·62 (134·77)	–4·97 % (27·13 %)	82·89 (105·98)	17·73 % (21·01 %)	29·41 %
Restaurants (*n* 76)	660·67 (318·30)	–21·97 (175·63)	–8·71 % (31·73 %)	123·30 (126·22)	21·38 % (24·96 %)	35·53 %
Entertainment (*n* 14)	552·79 (330·88)	63·14 (204·14)	1·91 % (43·10 %)	119·50 (174·89)	22·71 % (35·88 %)	28·57 %
Item category						
Starter (*n* 52)	342·85 (220·37)	–17·61 (127·99)	–19·42 (55·76)	84·88 (96·71)	33·44 % (48·60 %)	48·08 %
Main (*n* 59)	763·98 (360·26)	6·10 (184·10)	–3·77 (22·36)	122·21 (136·88)	16·15 % (15·84 %)	28·81 %
Dessert (*n* 60)	520·73 (190·61)	–31·23 (132·03)	–6·66 (28·66)	85·35 (104·95)	17·77 % (23·34 %)	23·33 %
Popular (*n* 57)	658·03 (330·78)	–26·45 (154·66)	–7·06 (27·33)	110·64 (110·33)	18·75 % (20·95 %)	35·09 %
Drink (*n* 8)	144·18 (84·81)	–0·57 (38·06)	–3·74 (30·19)	31·86 (16·99)	25·00 % (15·00 %)	75·00 %
Potentially inaccurate (*n* 59)^||^	738·16 (378·56)	–35·45 (169·36)	–9·93 % (31·56 %)	121·96 (121·78)	20·88 % (25·64 %)	35·59 %
Local authority						
Liverpool (*n* 119)	538·15 (298·88)	–27·03 (130·10)	–8·93 % (29·63 %)	87·51 (99·70)	19·62 % (23·91 %)	34·45 %
Milton Keynes (*n* 117)	587·77 (354·39)	–6·19 (166·28)	–8·56 % (40·33 %)	110·20 (124·26)	23·01 % (34·19 %)	33·33 %

* Negative values indicate that reported kcal were greater than measured.

† Percentage difference for reported – measured kcal.

‡ Mean difference between reported and measured kcal regardless of direction (– or +) of difference.

§ Numbers in brackets are sd.

|| Potentially inaccurate items not included in mean and standard deviation values for ‘overall’, ‘item category’ and ‘outlet category’.

In the main analyses (all menu items except for those that were potentially inaccurate), 56 % of items had a lower measured energy content than reported on menus. For over one-third (35 %) of items, the energy content reported on menus was outside of the 20 % leeway permitted; for 66 % of these (23 % of total), this was due to reported energy being substantially higher than measured and for 31 % of these (11 % of total) this was due to reported energy being substantially lower than measured. Findings were similar in the potentially inaccurate items (see online Supplementary Material 4).

**Figure 3.**
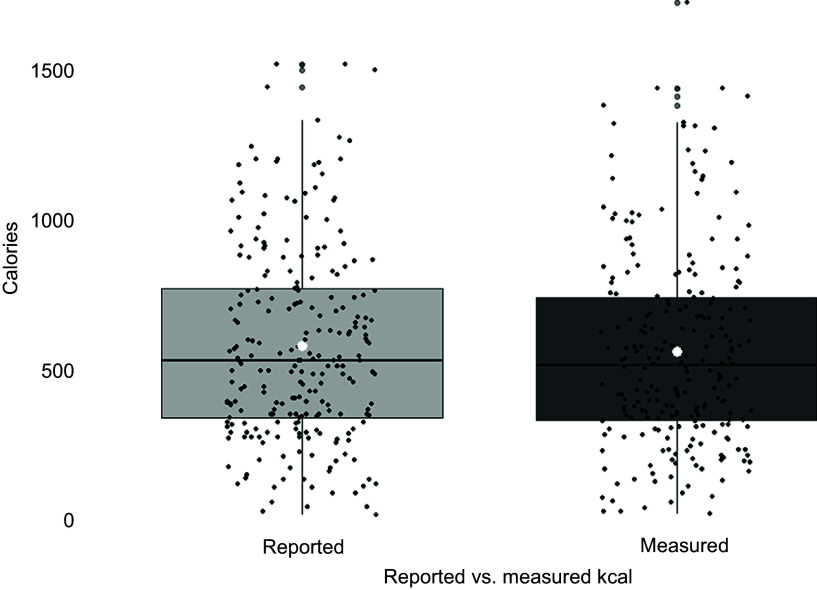
Reported and measured energy content of *n* 236 items. *white circle indicates the mean, ** outliers are shown twice (see grey points).

Two Wilcoxon signed rank tests found that the absolute percentage difference between reported and measured energy (*n* 236) was significantly greater than 0 % (*V* = 27 261, *P* < 0·001) but not greater than 20 % (*V* = 0, *P* > 0·99). However, reported and measured energy content were significantly different from each other, whereby on average energy content reported on menus was significantly greater than measured energy content (*V* = 16 920, *P* < 0·01, *r* = 0·182). A boxplot of mean differences is shown in Figure 3. Reported and measured energy were not significantly different for potentially inaccurate items.

**Figure 4.**
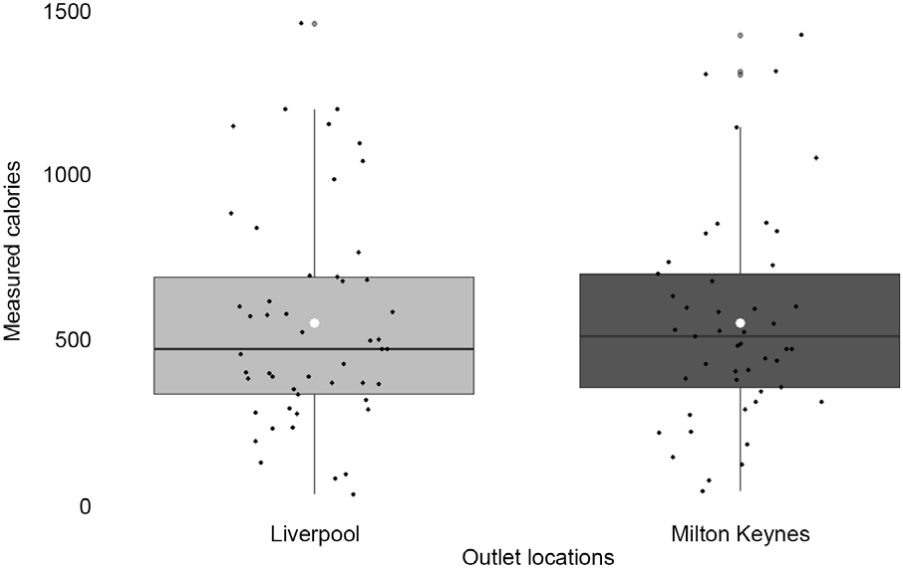
Matched pairs of menu items across the two local authorities (*n* 50 matched pairs). *white circle indicates the mean.

For the subsample of menu items from outlets matched across the two LA, a Wilcoxon signed rank test found no significant differences in measured energy between matched items (V = 583, *P* = 0·775), see Figure 4. The mean energy content of these items in Liverpool was 547·39 kcal ± 327·96 and for Milton Keynes this was 549·45 kcal ±312·02. The mean difference (Liverpool – Milton Keynes) was −14·15 kcal, and the relative percentage difference was −7·99 %. 73 % of items sampled in Milton Keynes were within 20 % of the value in Liverpool.

A linear model explored predictors of the mean difference between reported and measured energy content (Table 2). Variance inflation factor for all predictors were below 2, indicating minimal multicollinearity. Items with a higher measured energy content were more likely to have a measured energy higher than reported energy (B = 0·22, 99 % CI 0·12, 0·32, *P* < 0·001), whereby every measured 1 kcal increase was associated with an increased difference of 0·22 kcal. Main effects were also observed for outlet type and item category, whereby pubs and restaurants (*v*. cafes) and popular dishes (*v*. starters/sides) had a greater difference between reported and measured energy. There were no significant predictors of difference between measured and reported energy for potentially inaccurate items (full results in online Supplementary Material 4). We also explored predictors of absolute mean percentage difference between reported and measured energy content, and results were similar to main analyses (online Supplementary Material 5).

**Table 2.** Linear model exploring predictors of mean relative difference between reported and measured energy content (calculated as measured – reported)

	Mean kcal difference		
Predictors	Estimates	99 % CI	*P*
(Intercept)	–16·87	–111·00, 77·27	0·642
Pub*	–134·08	–222·36, −45·80	**< 0·001**
Fast Food*	–61·09	–144·82, 22·64	0·059
Restaurant*	–99·88	–185·36, −14·39	**0·003**
Entertainment*	8·66	–111·15, 128·47	0·851
Main^†^	–79·16	–161·72, 3·39	0·013
Dessert^†^	–64·15	–136·38, 8·08	0·022
Popular^†^	–88·89	–166·98, −10·79	**0·003**
Drink^†^	–22·60	–174·43, 129·23	0·699
IMD quintile [2]^‡^	–5·59	–84·61, 73·43	0·854
IMD quintile [3]^‡^	3·08	–55·14, 61·30	0·891
IMD quintile [4]^‡^	3·75	–84·58, 92·08	0·912
IMD quintile [5]^‡^	–54·97	–246·44, 136·50	0·456
Outlet location [MK]^§^	15·66	–34·90, 66·21	0·422
Total energy kcal	0·22	0·12, 0·32	**< 0·001**
Observations	236		
R^2^/R^2^ adjusted	0·181/0·129		

IMD, index of multiple deprivation.

Reference categories are *Cafes, ^†^Starters/sides, ^‡^IMD quintile 1, ^§^Liverpool.

Negative values indicate a decreased relative difference.

Bold text indicates significant *P* value.

To explore identified significant predictors, paired samples tests (Wilcoxon signed ranks) were conducted that examined differences between measured *v*. reported energy for cafes, restaurants and pubs and the starter and popular item categories. Pubs were the only outlet type where reported and measured energy were significantly different from each other (V = 943, *P* < 0·01). For pubs, mean reported kcal were 725·00 ± 326·89 and mean measured kcal were 674·10 ± 338·36. There was no significant difference between measured and reported energy for the starter or popular menu categories. Mean differences, percentage differences and the proportion of items outside of the 20 % leeway for each outlet type and item category are shown in Table 1.

## Discussion

This study of large business owned OOH food outlets subject to the mandatory calorie labelling law in England during 2024 found that averaged across all sampled menu items, the energy content (kcal) reported on OOH outlet menus was significantly greater than measured energy. However, it was common for reported energy content of menu items to be greater and less than measured energy content. For 35 % of menu items, reported energy was over 20 % greater or less than measured energy. Pubs had a particularly pronounced difference between reported and measured energy content compared with other outlet types. The measured energy content of the same menu items sampled from different outlets of the same chain were largely similar. Collectively, these findings suggest that reported energy content for significant numbers of menu items in the OOH sector in England differ substantially to measured energy content.

Expressed as a relative calorie value and a percentage of each item’s measured energy content, average reported energy of items was 17 kcal and 9 % greater than measured energy content. However, expressed as an absolute value (size of deviation from measured energy content irrespective of direction), the mean difference between measured and reported energy was 99 kcal and 21 %, due to both over- and under-estimation of measured energy content being common (although over-estimation of energy content was observed more frequently). This suggests that on average, calories reported for menu items in the English OOH food sector may differ substantially to their measured energy content. The overall pattern of results differs somewhat to research conducted in the USA^(22)^ which identified that measured energy content tended to be greater than reported on labelling and on restaurant menus. This difference in our findings may be a result of differences in types of outlets or menu items examined. Previous research has largely sampled packaged foods, which may be less prone to variability during the preparation and cooking process compared with items made in the OOH food sector. There may also be differences in the sampling methods used that could impact the measured energy content of items (e.g. sampling and packaging hot food *v*. sampling cold, packaged foods). One USA study considered OOH foods, but limited the investigation to menu items under 500 kcal^(22)^, which may help to explain differences as our findings would suggest there is less scope for inaccuracy at lower levels of energy content. However, our findings are comparable to those of a study conducted in Canada sampling a range of outlet types^(21)^, whereby items had an overall greater mean labelled energy content than mean measured energy content, with evidence of both over and under-estimation of reported energy content.

In the previous North American studies discussed, reported energy content was largely within the 20 % leeway of measured content (70 %^(20)^, 86 %^(21)^, 59 %^(22)^), and this is comparable to the present study (65 %). However, across all of these and the present study, a proportion of menu item labels appears to be significantly different from measured values (> 20 %). There are multiple possible explanations for the observed error in reported energy content in the present and prior research, for example, errors in ingredient information or human error in calculation. It would now be informative to study what the causes of inaccuracy are. In England, the calorie labelling legislation outlines several accepted methods to calculate energy content, and for this research we chose to use the gold standard of measurement (i.e. bomb calorimetry)^(27)^. It is plausible that the observed error comes from businesses utilising alternative methods (e.g. calculation using standardised ingredient information) to estimate energy content^(5)^.

While five different outlet types were explored, the largest difference between mean reported and measured energy content was observed for pubs. This outlet type had 46 % of items outside of the 20 % legislation leeway, while all other outlet types had between 30 and 36 % of items outside of the leeway. Observed differences between measured and reported energy may have been greater in pubs due to differences in the use of ingredients that are more prone to fluctuations in water and energy content through cooking. This, along with potentially greater autonomy for chefs in how foods are prepped and cooked (e.g. compared with fast food outlets or the café chains sampled, where many food ingredients are pre-packaged, and serving sizes more standardised), may explain why discrepancies were greater in pubs. Alternatively, differences between outlet types and individual outlets may exist if there are differences in how the energy content of menu items is calculated.

When items deemed potentially inaccurate were examined, results were similar to the main analysis. Items in this category were selected based on two researcher’s assessments of all items on a menu with consideration of the description and composition of the dish and the potential for inconsistencies in portion sizes made by servers in outlets. As these findings were not largely different from the items in the main analyses, this suggests that reliably identifying likely inaccurately labelled menu items based on menu information alone may be difficult.

Our findings highlight the difficulty that enforcers of the policy may have if attempting to assess accuracy of calorie labelling in OOH outlets without expending substantial resources on laboratory measurement. At present there appears to be minimal enforcement training supplied, although how enforcement is monitored is at the discretion of the LA^(5)^. To aid improved accuracy of a menu’s calorie information and enforcement of the policy, laboratory analysis of menu items would preferably determine reported energy content; however, this would result in significant financial cost to businesses. Despite this, a one-off or annual cost may be minimal for larger food chains such as those explored in this study. Greater support for businesses in calculating energy content, as well as greater training and assistance for the individuals responsible for assessing compliance, would likely help to reduce the discrepancy between measured and reported energy content. Additionally, clearer pathways for compliance monitoring and sanctions for observed inaccuracy may be necessary to improve accuracy of labelling by businesses. Our results showed that the same menu items from different outlet locations of the same business tended to have similar measured calorie content, so analysis at the chain level is likely to be somewhat representative of all outlets in a chain. However, this should be explored in greater depth, and particularly within pubs, where the greatest inconsistencies in reported *v*. measured energy content were observed.

### Strengths and limitations

There are a number of limitations of this work that should be considered alongside findings. This study explored a large sample of menu items in the OOH food sector; however, this sample is not representative of all menu items in outlets in England and instead provides a snapshot of the accuracy of calorie labels in the OOH food sector among large businesses. The analyses relating to matched samples and exploration of potentially inaccurate items can be considered as exploratory only, as the study was not powered for these smaller samples. We used a gold standard measurement of energy content, and this is a strength of the present work. However, we collected one sample per menu item from outlets for our main analyses, and future research would benefit from taking multiple samples of the same item. This would improve measurement accuracy for estimated energy content and also allow for examination of consistency of menu item energy content within the same outlet.

### Conclusion

There were significant inaccuracies in reported energy content of calorie labelled menu items in English food outlets subject to mandatory calorie labelling, and this appears to be caused by both over- and under-estimation of reported energy content.

## Supporting information

Finlay et al. supplementary material Finlay et al. supplementary material

## Supplementary material

For supplementary material/s referred to in this article, please visit https://doi.org/10.1017/S0007114525105217
